# Effect of CeO_2_ on Microstructure and Wear Resistance of TiC Bioinert Coatings on Ti6Al4V Alloy by Laser Cladding

**DOI:** 10.3390/ma11010058

**Published:** 2017-12-31

**Authors:** Tao Chen, Defu Liu, Fan Wu, Haojun Wang

**Affiliations:** 1 College of Mechanical and Electrical Engineering, Central South University, Changsha 410083, China; chent0731@csu.edu.cn (T.C.); wufan1993@csu.edu.cn (F.W.); wanghaojun@csu.edu.cn (H.W.); 2 State Key Laboratory of High Performance Complex Manufacturing, Changsha 410083, China

**Keywords:** TiC bioinert coatings, laser cladding, CeO_2_, microstructure, wear resistance

## Abstract

To solve the lack of wear resistance of titanium alloys for use in biological applications, various prepared coatings on titanium alloys are often used as wear-resistant materials. In this paper, TiC bioinert coatings were fabricated on Ti6Al4V by laser cladding using mixed TiC and ZrO_2_ powders as the basic pre-placed materials. A certain amount of CeO_2_ powder was also added to the pre-placed powders to further improve the properties of the TiC coatings. The effects of CeO_2_ additive on the phase constituents, microstructures and wear resistance of the TiC coatings were researched in detail. Although the effect of CeO_2_ on the phase constituents of the coatings was slight, it had a significant effect on the microstructure and wear resistance of the coatings. The crystalline grains in the TiC coatings, observed by a scanning electron microscope (SEM), were refined due to the effect of the CeO_2_. With the increase of CeO_2_ additive content in the pre-placed powders, finer and more compact dendrites led to improvement of the micro-hardness and wear resistance of the TiC coatings. Also, 5 wt % content of CeO_2_ additive in the pre-placed powders was the best choice for improving the wear properties of the TiC coatings.

## 1. Introduction

Titanium (Ti) and its alloys are widely used in biomedical fields. Compared with other biomaterials such as stainless steels, cobalt-based alloys and tantalum (Ta), Ti and its alloys have excellent material characteristics such as low modulus and density, high specific strength, good fatigue strength, corrosion resistance and biocompatibility [1,2]. However, the clinical application of titanium alloys for hard-tissue replacements and/or intensive wear applications is seriously limited due to their low hardness and poor wear resistance [3,4].

In order to maintain excellent quality in titanium alloys and improve the tribological properties of their surface, various surface modification techniques are employed, such as plasma spraying [5,6], micro-arc oxidation [7], physical vapor deposition (PVD) [8], electrochemical anodization [9] and laser cladding [10,11]. For instances, Khanna et al. fabricated a thin layer of α-alumina with a thickness of 30 μm on a Ti6Al4V substrate by a methodology that included cold spraying, heat treatment and micro-arc oxidation, and the result showed that the micro-hardness of the layer was comparable to that of the monolithic alumina used on the femoral head [12]. Shtansky et al. reported that CaO- and ZrO_2_-doped TiCxNy coatings were fabricated by self-propagating high-temperature synthesis, which had not only a low friction coefficient and high wear resistance, but also biocompatibility and non-toxicity [13]. Yusuf Kayali fabricated the TiN and TiAlN coatings by PVD, with the result that the wear resistance of the coatings were 6 and 15 times higher than that of uncoated substrates, respectively [14]. Compared with laser cladding, however, the coatings produced by plasma spraying and other surface modification techniques cause some problems such as thin, multiple cracks and poor adhesion between the coatings and substrates [15]. The bond strength between the coating layer and the metal substrate is crucial; separation of the coating layer from the implant during service in the human body means detached particles can be unfavorable to body health and surrounding tissue [16]. According to published reports, laser cladding is a promising and effective surface modification technology.

Laser cladding is a hard-facing process that uses a high-power laser beam to melt the preplaced powder materials and a thin layer of the substrate to form a pore- and crack-free coating 50 μm–2 mm thick with low dilution that is perfectly metallurgically bonded to the substrate [17]. Some researchers have carried out relevant work on laser-cladding processes in the biomedical field. The typical hydroxyapatite (HA) coatings were fabricated on Ti alloy substrate by laser cladding [18,19]. Liu et al. and Zheng et al. fabricated the calcium phosphate coatings with bioactivity on Ti6Al4V [20,21]. Wang et al. fabricated the CaO–SiO_2_ coatings using wollastonite (CaSiO_3_) powder on Ti6Al4V [22]. Tsung-Yuan Kuo et al. reported that Al_2_O_3_ reinforced fluorapatite (FA) coatings were fabricated on Ti6Al4V by laser cladding and, as a result, the coatings exhibited excellent bioactivity and their hardness was significantly improved [23]. Although the bioactive coatings represented by HA was deposited on the metal alloy to assist the osseointegration of these implants with surrounding tissues [24], which could also improve the mechanical properties of the metal such as its load-bearing ability [25], this kind of coating was susceptible to fatigue failure, making it unsuitable for load-bearing and wear-resistant implants such as articular surfaces [26,27]. However, it has rarely been reported that bioinert ceramic coatings with a better wear resistance can be prepared by laser cladding on Ti alloys.

According to the published reports, alumina (Al_2_O_3_), zirconia (ZrO_2_), titaniumcarbide (TiC) and titaniumnitride (TiN) coatings triggered no adverse effects on cells in culture. Meanwhile, the fibroblasts showed a rapid and logarithmically stable growth curve on them [28,29]. Combined with the previous reports, these are the promising materials for making wear-resistant coatings in biomedical applications such as orthopaedic implants. Among them, TiC has a high hardness, as well as a higher melting point and absorption of laser energy [30,31]. These characteristics of TiC materials are beneficial for fabricating coatings by laser-cladding processes. Meanwhile, ZrO_2_ powder is introduced into TiC coatings to improve the mechanical properties of the coatings. Chien et al. fabricated fluorapatite (FA)/zirconia composite coatings by laser cladding using FA and 20 wt % yttria (3 mol %) stabilized zirconia (ZrO_2_, 3Y-TZP) [11]. Although the ZrO_2_ was completely decomposed during the cladding process, the properties of the coatings were still enhanced. Meanwhile, the hardness of this coatings was higher than the FA coatings fabricated by laser cladding using pure FA under similar laser source and cladding parameters [32], which was attributed to the addition of ZrO_2_ to the FA matrix. In addition, the appropriate additive of rare earth oxides can improve the mechanical properties of the coatings [33]. Therefore, in this work, wear-resistant coatings are fabricated by laser cladding using mixed TiC, ZrO_2_ and CeO_2_ powders. The effects of CeO_2_ on phase constitution, microstructure, micro-hardness and wear resistance of the laser-cladding coatings are investigated in detail. We expect that the selected materials and methods in the paper can be used for the manufacture of the load-bearing and wear-resistant surfaces of artificial joints in the future.

## 2. Experimental Procedures

### 2.1. Materials Used

Ti6Al4V plate specimens (size: 30 mm × 35 mm × 6 mm) were used as the substrate material. The chemical composition (wt %) of the Ti6Al4V substrate was C 0.01, N 0.01, Fe 0.05, V 4.06, Al 6.37 and Ti 89.5. The substrate surfaces were first ground with 600 grit abrasive papers to remove oxidation layers and then ultrasonically cleaned with acetone prior to laser cladding. The preplaced powder contained TiC (≥99.5% purity, 50 nm), ZrO_2_ (≥99.5% purity, 30 nm), CeO_2_ (≥99.5% purity, 20 nm), whose ingredients (wt %) are shown in Table 1. The 20% (wt %) ZrO_2_ was added to the TiC matrix in order to further improve the mechanical properties of the coatings [11,32]. According to the contents of CeO_2_ 0 wt %, 1 wt %, 3 wt %, 5 wt % and 7 wt %, the mixed powders were marked as 0Ce, 1Ce, 3Ce, 5Ce and 7Ce, respectively. The powder mixtures were mixed for 2 h at a rotational speed of 200 r/min in a horizontal ball miller. The mixed powders were pre-placed on to the surfaces of the substrates with organic binder at a thickness of approximately 0.4 mm.

### 2.2. Laser-Cladding Process

**Figure 1:**
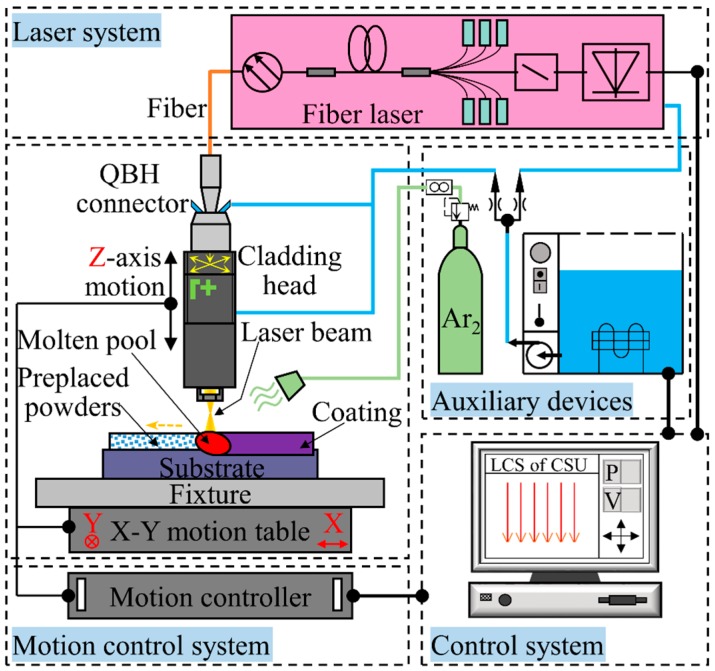
Schematic illustration of the laser-cladding system.

The laser-cladding experiments were carried out on the laser-cladding set-up designed and assembled by ourselves, as shown in Figure 1. Core features of this laser-cladding system include the laser system, the motion-control system, the computer-control system and the auxiliary devices. The laser system consisted of two parts: the fiber laser system and the optical focusing system. The fiber laser system as a heat source was a RFL-C500 medium-power single mode CW fiber laser (Wuhan Raycus Fiber Laser Technologies Co., Ltd., Wuhan, China) with a maximum output power of 500 W. The laser-cladding head (RayTools AG, Burgdorf, Switzerland) integrated a lens with a diameter of 75 mm and a focal length of 250 mm employed in the optical focusing system. A positive defocus was used to obtain the spot with a desired diameter. The fiber laser and the laser-cladding head were joined by a QBH standard connector. The motion table and laser-cladding parameters were controlled by a computer-control system. When laser-cladding experiments were conducted, the laser beam energy melted rapidly both the pre-placed powders and a thin layer of the substrates [34]. At the same time, as the motion table moved, the melted material solidified rapidly, and then a continuous cladding coating formed on the surface of the substrate. The selected process parameters of the laser-cladding experiments in this paper were as follows: laser output power 200 W, laser beam spot diameter 1 mm, laser beam scanning speed 5 mm/s, overlapping ratio 20%. In addition, Ar_2_ shielding gas of 10 L/min was used to protect the melt pool from oxidation.

### 2.3. Microanalysis of the Coatings

To obtain the cross-section morphologies and microstructures of the coatings, the samples were cut by an electro-sparking machining, and ground with SiC grit paper, then polished with 0.05 µm Al_2_O_3_ finish, and etched by Kroll’s reagent (HF: 2%; HNO_3_: 5%; and water: balance) for 20 seconds. The cross-section morphologies and microstructures of the coatings were examined by a MIRA3 TESCAN scanning electron microscope (SEM, TESCAN Co., Brno, CZE) coupled with energy-dispersive spectroscopy (EDS, Oxford Inc., Oxford, UK). The phase compositions of the coatings were identified by a Rigaku D/max 2500 X-ray diffractor (XRD, Bruker, Berne, Switzerland) using 60 kV, 40 mA and Cu K_α_ radiation operated in a scanning range of 2θ from 20° to 80°.

### 2.4. Micro-Hardness and Wear Resistance

Micro-hardness distributions of the coatings were measured by an automatic micro-hardness tester (HVS-1000Z, Vegour, Shanghai, China) with a testing load of 200 g and dwelling time of 15 s.

**Figure 2:**
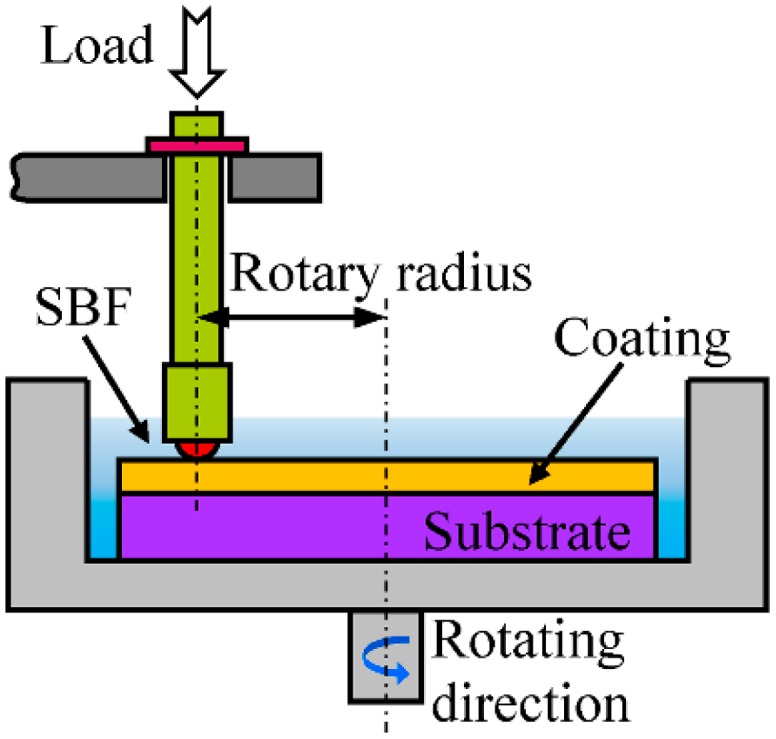
Schematic diagram of friction and wear test.

As shown in Figure 2, the wear properties of the coatings and the substrate were tested on a ball-on-disk tribo-meter (HT-1000 tester, Zhongkekaihua science and technology Co., Ltd., Lanzhou, China) under simulated body fluid (SBF) lubrication conditions at an ambient environment against a φ4 mm Si_3_N_4_ ceramic ball. The test parameters are listed in Table 2. At least 3 duplicates of the wear test were run for each test condition. To investigate the wear mechanism, the worn morphologies of the samples were also observed by SEM. Before wear test, the test samples were ground with 120, 240, 600 and 1000# SiC grit paper, respectively. The section profiles of wear track and wear areas area were measured by a digital microscope (VHX-5000, KEYENCE, Osaka, Japan). The wear volume loss (*V*) is calculated using Equation (1).

(1) V=2πr·A

where *r* represents the radius of wear track, and *A* is the wear areas of the wear track.

The wear rate is calculated using the following Equation (2).

(2) Wear rate=V/T

where *V* is total wear volume, and *T* is the wear time.

## 3. Results and Discussion

### 3.1. Phases of the Coatings Fabricated by Laser Cladding

**Figure 3:**
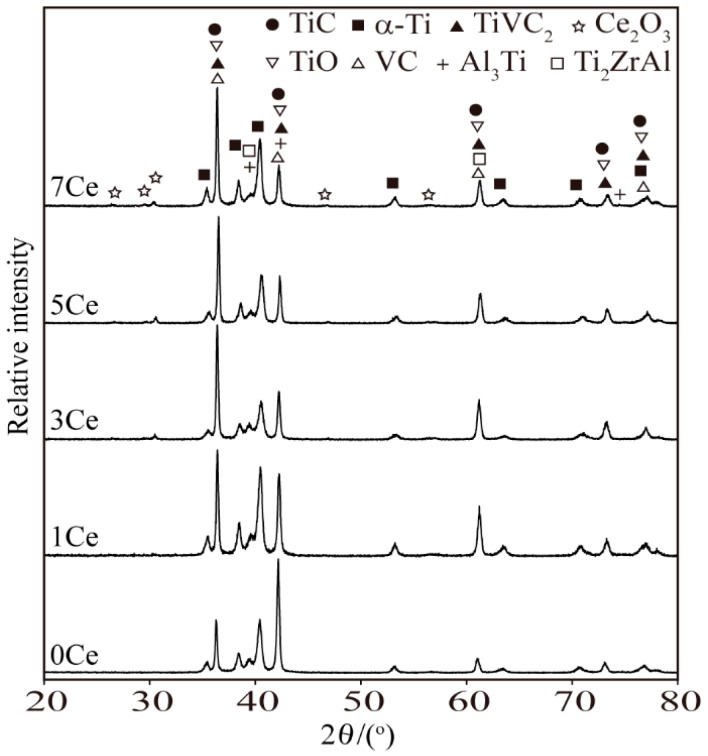
X-ray diffraction (XRD) spectrums of the coatings.

The phases of the TiC coatings fabricated by laser-cladding processes using the pre-placed powders with different contents of CeO_2_ additive are presented in Figure 3. It can be seen that the coating fabricated by the pre-placed powders without CeO_2_ additive was mainly composed of TiC, α-Ti, TiO, VC, TiVC_2_, Al_3_Ti and Ti_2_ZrAl phases. With the increase of CeO_2_ additive in the pre-placed powders, the diffraction peaks of Ce_2_O_3_ appear in the diffraction patterns when 3 wt % and above CeO_2_ additive is added. However, the diffraction peaks of Ce_2_O_3_ do not appear in the coating with 1 wt % CeO_2_ additive. It should be noted in patricular that the diffraction peaks of ZrO_2_ and CeO_2_ are not detected in the coatings.

During the laser-cladding process, the preplaced powders and the surface layer of the Ti6Al4V substrate simultaneously melt, and thus a large amount of Ti released from the substrate enters into the molten pool due to the dilution effect. Meanwhile, small amounts of the V element and Al element from the substrate also enter into the molten pool. Complex chemical reactions then occur in the molten pool, leading to the formation of the various phases mentioned above. There are two possible sources of the TiC phase: one is the unfused original TiC ceramic particles, the other is the second precipitated phase. Ther TiC has high hardness, which is beneficial to the mechanical properties of the coatings; therefore, it has been reported that TiC-reinforced metal matrix composite coatings offer significant progress in terms of average micro-hardness and wear resistance [35,36].

It is noted that the original ZrO_2_ and CeO_2_ are not detected in the coatings, which should be attributed to a sufficient chemical reaction during the laser-cladding procedure. In fact, the following Equations (3)–(6) describe the different reactions between TiC and ZrO_2_, reported by V. M. Gropyanov [37,38]:

(3) $$\mathrm{TiC}+2{\mathrm{ZrO}}_{2}\to \mathrm{TiO}+2\mathrm{ZrO}+\mathrm{CO}↑$$

(4) $$\mathrm{TiC}+{\mathrm{ZrO}}_{2}\to \mathrm{Ti}+\mathrm{ZrO}+\mathrm{CO}↑$$

(5) $$\mathrm{TiC}+{\mathrm{ZrO}}_{2}\to \mathrm{TiO}+\mathrm{Zr}+\mathrm{CO}↑$$

(6) $$2\mathrm{TiC}+{\mathrm{ZrO}}_{2}\to 2\mathrm{Ti}+\mathrm{Zr}+2\mathrm{CO}↑$$

As a result, TiO, ZrO, Ti and Zr were generated in the molten pool. Among them, ZrO and Zr are in a liquid gas state, which accounted for low content of Zr element in the coatings. TiO and TiC have an isotypic lattice structure and the same lattice parameter, which leads to the formation of a continuous solid solution between them. These factors are beneficial to the mechanical properties of the coatings. In addition, it was proved that the coatings have the Ce element because the Ce_2_O_3_ phase was detected. The Ce_2_O_3_ phase appeared in the coatings because of chemical reactions between CeO_2_ and CO; namely, CeO_2_ is reduced by CO with strong reducibility. According to the report of Jesús Graciani [39], the chemical reaction is described as follows:

(3) $$4{\mathrm{CeO}}_{2}+2\mathrm{CO}\to 2{\mathrm{Ce}}_{2}O_{3}+2{\mathrm{CO}}_{2}↑$$

In addition, it has been reported that chemical reactions could occur between CeO_2_ and Ce atoms from partially decomposed CeO_2_/Ce_2_O_3_ at high temperatures [40]:

(4) $$3{\mathrm{CeO}}_{2}+\mathrm{Ce}\to 2{\mathrm{Ce}}_{2}O_{3}$$

On the other hand, the V element is a kind of strong carbide-forming element, and its carbide phases such as in situ synthesized VC are fine, so the movement of the grain boundary can be prevented and the microstructure of the coatings is fine [41]. Moreover, in situ synthesized VC can further react with TiC, forming TiVC_2_. It has also been previously reported that in situ synthesized VC and TiVC_2_-reinforced Fe-based coatings have been fabricated by laser cladding [42].

In summary, according to the results of XRD analysis, the phase types of the coatings fabricated by pre-placed powders with different content of CeO_2_ additive display no obvious change. The coatings are mainly composed of TiC, TiO, VC, TiVC_2_, α-Ti and a little in situ synthesized intermetallic compounds, which are propitious for the hardness and wear resistance of the coatings.

### 3.2. Microstructures of the Coatings

**Figure 4:**
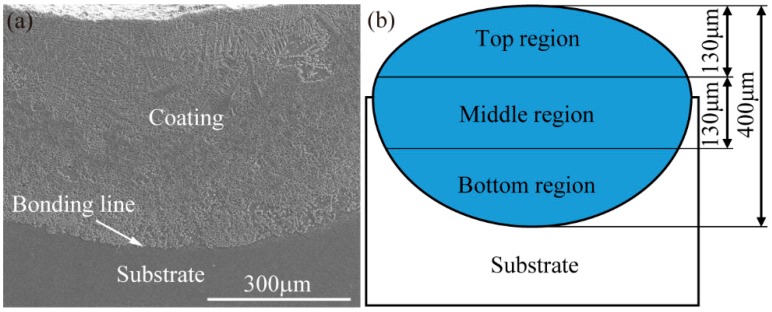
(**a**) Typical macrostructure of the coating; (**b**) 3 regions of the coating.

The cross-section macrostructure of the 3Ce coating is presented in Figure 4a, which is typical for all the cladding coatings. The coating and substrate can be clearly observed, and the metallurgical bond is formed between them. In order to investigate the evolution of the microstructures of the coatings’ top surface to its inside, cross-section morphologies of the coatings should be observed by SEM. Therefore, the cross-section of the coatings were divided into 3 regions from the top surface to inside: the top region (0–130 μm), the middle region (130–260 μm), and the bottom region (260–400 μm), as shown in Figure 4b.

**Figure 5:**
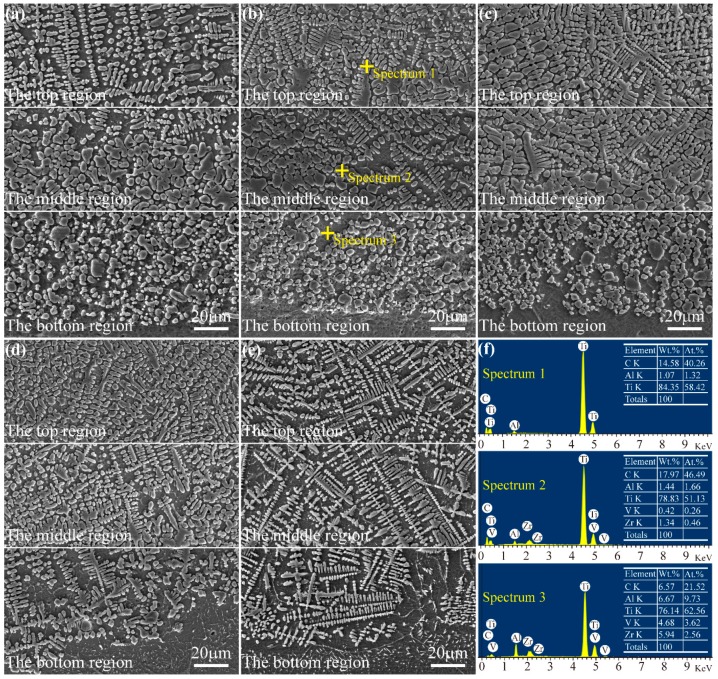
The cross-sectional morphologies of laser-cladding coatings (**a**) without CeO_2_; (**b**) 1% CeO_2_; (**c**) 3% CeO_2_; (**d**) 5% CeO_2_; (**e**) 7% CeO_2_; (**f**) corresponding energy-dispersive spectroscopy (EDS) result of a coating with 1% CeO_2_.

The morphologies of the different cross-sectional regions of the coatings are shown in Figure 5. Figure 5a presents the cross-sectional morphologies of the coating fabricated by the pre-placed powders without CeO_2_ additive. From this figure, it can be seen that the microstructure of the 0Ce coating is mainly dendrites in the upper region, while granular crystals are in the bottom region. It has been reported that the solidification morphology in each region in a laser-cladding process mainly depends on the solid/liquid interface stability factor that is the ratio of the temperature gradient (*G*) to the solidification rate (*R*) [43,44]. When the technological parameters of the laser-cladding process are not changed, the solidification rate (*R*) gradually reduces from the top surface to inside the laser molten pool, while the temperature gradient (*G*) reverses. Therefore, the ratio of the temperature gradient to the solidification rate (*G*/*R*) increases in the laser molten pool from the top surface to inside, which gives rise to change on the microstructures of the coatings; granular crystals in the bottom region gradually change to dendrites in the top region. The crystal transformation of the coating is clearly observed in the middle region from Figure 5a.

Figure 5b–d show the cross-section morphologies of the coatings fabricated by the pre-placed powders with 1, 3, 5% CeO_2_ additives, respectively. Compared with the microstructure of 0Ce coating, the dendrites and granular crystals become more compact and finer with the increase of CeO_2_ additive in the pre-placed powders. Meanwhile, it can be seen that the size and distribution of dendrite microstructure on the 5Ce coating are more uniform, which is beneficial to the hardness and wear resistance of the coating. As shown in Figure 5d,e, the microstructures of the 5Ce and 7Ce coatings in the bottom region become dendrites, which may be attributed to the reduced temperature gradient. Among them, the dendrites of the 7Ce coating are different from those of other coatings; it can be observed from the figure that the dendrites of the 7Ce coating are not compact, and secondary dendrite arm spacings increase significantly, which may be attributed to the fact that excessive CeO_2_ could result in an even lower temperature of the molten pool. According to relevant reports [45,46], the crystalline grain-refinement effect might be related to the content of rare earth oxide, and excessive rare earth oxide would reduce the function of refinement. Therefore, only an appropriate amount of CeO_2_ additive in the pre-placed powders could refine the crystalline grain of the coatings. In addition, combined with the results of X-ray diffraction and EDS, the composition of typical microstructure on the coatings fabricated by pre-placed powders with different content of CeO_2_ additive was analyzed. The results showed that the compositions of typical microstructure on the different coatings are similar, so just the 1Ce coating is taken as an example. As shown in Figure 5f, the dendrites and granular crystals are rich in Ti and C elements, and the atomic ratio between them are closer to 1:1, so the reinforced phase is considered as TiC (spectrum 1 and 2). Moreover the source of the TiC phase is identified as the second precipitated phase due to high laser-power density. According to the EDS result of spectrum 3, Al and V elements can be seen in the solid solution because partial Al and V element are diffused from the substrate into the coatings.

### 3.3. Micro-Hardness of the Coatings

**Figure 6:**
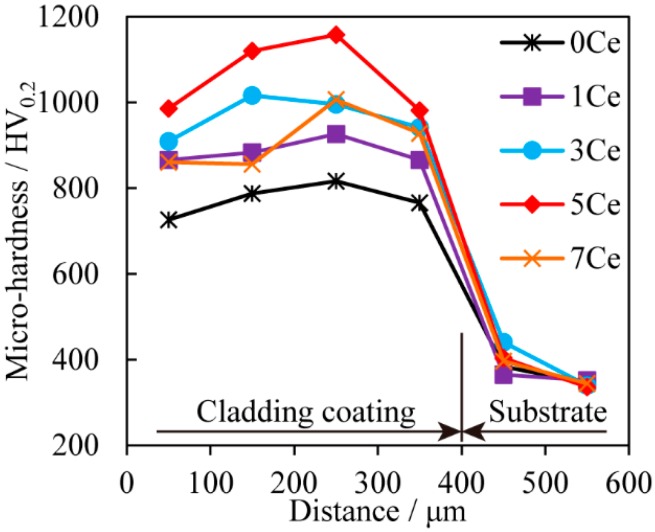
Micro-hardness distribution of the specimens.

The micro-hardness distributions along the depth direction of the coatings are shown in Figure 6. Each point in this figure is the average micro-hardness value of the coating obtained from at least 5 indentations. It can be observed that all the coatings have similar micro-hardness distributions. The coatings exhibit higher micro-hardness compared with the Ti6Al4V substrate, and the micro-hardness of the coatings fabricated using the pre-placed powders with CeO_2_ additive is further improved. The average micro-hardness of the 0Ce coating is about 2.3 times higher than that of the Ti6Al4V substrate (336.8 HV_0.2_). With the increase of CeO_2_ additive in the pre-placed powders, the average micro-hardness of the 1Ce, 3Ce, 5Ce and 7Ce coatings are enhanced by about 2.6, 2.9, 3.2 and 2.7 times than that of the Ti6Al4V substrate, respectively. It was found that 5Ce coating had the highest micro-hardness due to the crystalline grain-refinement effect of the CeO_2_ additive. According to the traditional Hall–Petch relationship [47], finer crystalline grain size is beneficial to the improvement of strength and toughness so that the fine-grain strengthening effect is generated. However, the average micro-hardness of the 7Ce coating was lower than that of the 5Ce coating. As mentioned above, the excessive rare earth oxide would reduce the function of refinement. In addition, it has been reported that excessive rare earth oxide could lead to the loss by burning of TiC ceramic particles and restrain the precipitation of the secondary TiC phase, which caused the micro-hardness of the cladding coatings to decrease [46]. To sum up, the micro-hardness of the TiC coatings can be improved by appropriate CeO_2_ additive in the pre-placed powders.

### 3.4. Wear Resistance of the Coatings

**Figure 7:**
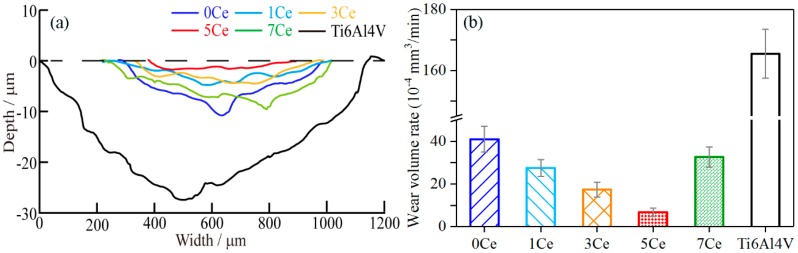
(**a**) The section profiles of a typical wear track; and (**b**) the wear volume rates of the substrate and the coatings.

The section profiles of the wear tracks of the Ti6Al4V substrate and the coatings fabricated by laser cladding are shown in Figure 7a. It can be seen from the figure that the substrate appears deeper with a wider wear track than that of the coatings; that is, the substrate has the largest wear extent, indicating poor tribological properties. Meanwhile, the wear degree of the coatings in wear-track depths and area values have distinctly decreased, indicating that the cladding coatings exhibit much better wear resistance than the substrate. It can be clearly observed from Figure 7a that the 5Ce coating has the minimum wear area; moreover, the wear areas of the 0Ce and 7Ce coatings are bigger than those of the 1Ce, 3Ce and 5Ce coatings.

The wear-volume rates of the substrate and the TiC coatings are presented in Figure 7b. The wear-volume rate of the Ti6Al4V substrate (165.47 × 10^−4^ mm^3^/min) is 4.04 times as big as that of 0Ce coating. When different contents of CeO_2_ were added in the pre-placed powders, the wear volume rates of the coatings appeared at different level underground falls compared with the 0Ce coating, which indicates that the CeO_2_ additive in the pre-placed powders was beneficial to wear resistance of the coatings. For the 1Ce, 3Ce, 5Ce and 7Ce coatings, the wear resistance was respectively increased by 1.48, 2.36, 6.08 and 1.25 times compared with that of the 0Ce coating. In other words, the 5Ce coating had the best wear resistance among these coatings, and its wear resistance was enhanced by 24.59 times that of the Ti6Al4V substrate. However, as the CeO_2_ additive content continued to increase, the wear resistance of the 7Ce coating declined significantly, which indicated that the excessive CeO_2_ in the pre-placed powders was not beneficial to the further improvement in wear resistance of the coatings. In summary, appropriate CeO_2_ additive in the pre-placed powders could enhance the wear resistance of the coatings.

**Figure 8:**
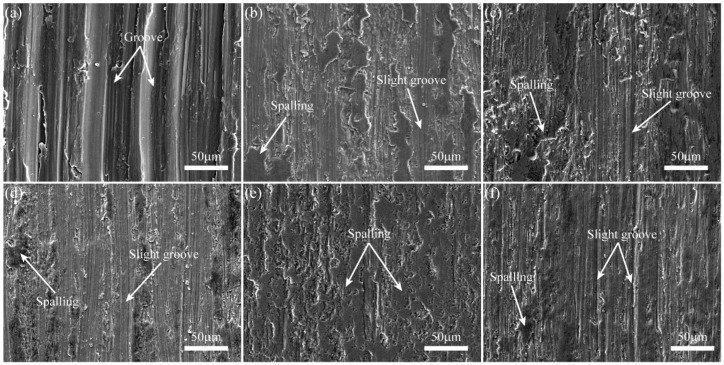
Typical worn surfaces of: (**a**) Ti6Al4V; (**b**) 0Ce; (**c**) 1Ce; (**d**) 3Ce; (**e**) 5Ce; (**f**) 7Ce coatings.

**Figure 9:**
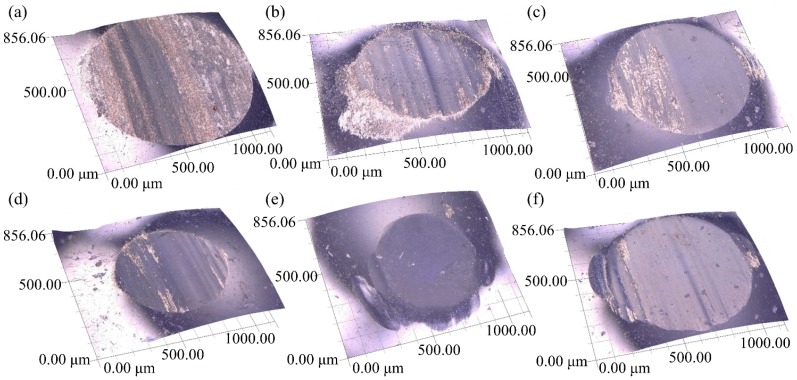
The worn surfaces of the corresponding counterbody balls of: (**a**) TiAl4V; (**b**) 0Ce; (**c**) 1Ce; (**d**) 3Ce; (**e**) 5Ce; (**f**) 7Ce coatings.

To understand the underlying wear mechanism of the coatings in simulated body fluid (SBF), the worn surfaces of the substrate and the coatings, as well as the worn surfaces of the corresponding Si_3_N_4_ ceramic balls, were respectively observed by SEM and digital microscope, as shown in Figure 8 and Figure 9. It can be observed from Figure 8a that the worn surface of the Ti6Al4V substrate has deep grooves and adhesive features, which indicates that the substrate suffered from severe abrasive and adhesive wear. During the wear test, it was easy for the hard asperities on the grinding ball to penetrate into the surface of the relatively soft titanium substrate, forming micro-cutting and deep grooves on the substrate surface. Moreover, the worn surface of the corresponding ceramic ball showed regular grooves and protuberances, as shown in Figure 9a. This result further confirms the existence of abrasive and adhesive wear between the friction pair. In contrast, the worn morphologies of the coatings did not have obvious characteristics of micro-cutting or ploughing grooves; meanwhile, the worn surface of the corresponding ceramic ball appeared to have irregular grooves and spalling, which should be attributed to two major reasons: one was the micro-hardness of the coatings were so high that the ploughing effects of the ceramic ball on the coatings was weakened; the other was that the spalling features were observed on the worn surfaces of the coatings due to alternating stress from the grinding ball. As shown in Figure 8b–d,f, the worn surfaces of the coatings appeared to have obvious spalling and slight grooves, which indicates that the wear mechanism of the coatings was adhesive wear and slight abrasive wear. It can be noted that the worn surfaces of 5Ce coating and the corresponding ceramic ball were the smoothest; the surface of the 5Ce coating had adhesive features and scarcely any ploughing grooves, indicating that adhesive wear played a major role in the 5Ce coating, as shown in Figure 8e and Figure 9e.

## 4. Conclusions

1 TiC coatings on Ti6Al4V alloy substrates were fabricated by the laser-cladding process with TiC–ZrO_2_–CeO_2_ mixed powders. The coatings were mainly composed of TiC, α-Ti, TiO, VC, TiVC_2_, Al_3_Ti and Ti_2_ZrAl phases.
2 The cross-sectional microstructures of the coatings from the top surface to the inside gradually changed from dendrites to granular crystals. With the increase of CeO_2_ additive content in the pre-placed powders, the microstructures of the coatings became more compact and finer. Excessive CeO_2_ additive in the preplaced powders led to a reduction in the function of refinement, as in the 7Ce coating.
3 The effect of CeO_2_ additive in the pre-placed powders on the mechanical properties of the coatings was obvious. The micro-hardness and wear resistance of the coatings increased with the increase of CeO_2_ additive content, but excessive CeO_2_ additive reduced the strengthening effect. Compared with the substrate, the micro-hardness and wear resistance of the 5Ce coating were improved by 3.2 and 24.59 times, respectively.

## Figures and Tables

**Table 1:** The ingredients of the pre-placed powder.

Powder Ingredient (wt %)			Number Marked
TiC	ZrO_2_	CeO_2_	
80	20	0	0Ce
79		1	1Ce
77		3	3Ce
75		5	5Ce
73		7	7Ce

**Table 2:** Experimental parameters of wear test.

Parameter	Value	Unit
Load	10	N
Temperature	25 ± 1	°C
Wear time	30	min
Rotation radius	5	mm
Rotation speed	200	rad/min
Solution	SBF	-
